# Effect of sodium-glucose Co-transporter 2 inhibitors on coronary microcirculation

**DOI:** 10.3389/fphar.2025.1523727

**Published:** 2025-02-28

**Authors:** Shaoxin Chen, Weiqian Ou, Shuguang Gan, Lixian Chen, Baohua Liu, Zhenhong Zhang

**Affiliations:** The Second People’s Hospital of Foshan, Foshan, China

**Keywords:** sodium-glucose Co-transporter 2 inhibitors, coronary microvascular disease, heart failure, SGLT2 inhibitor, HFpEF, heart failure with preserved ejection fraction

## Abstract

Coronary microvascular disease (CMVD) has emerged as a new target for the occurrence and development of heart failure treatment. Various indicators such as Index of Microvascular Resistance, Coronary Flow Reserve, Microvascular Resistance Reserve, Hyperemic Microvascular Resistance and Coronary Flow Velocity Reserve can be used to assess CMVD. Coronary microcirculation dysfunction is one of the important pathogenic mechanisms of heart failure. Sodium-Glucose Co-Transporter 2 (SGLT2) Inhibitors have been widely used in the treatment of various types of heart failure, but their specific pharmacological mechanisms are not yet fully understood. Studies have shown that SGLT2 inhibitors may be involved in the pathophysiology of CMVD by regulating cellular pathophysiological processes such as oxidative stress, mitochondrial function, energy metabolism, vascular genesis, and signalling pathways. Therefore, coronary microvascular dysfunction may be one of the treatment targets of using SGLT2 inhibitors in heart failure. Several animal experiments have found that SGLT2 inhibitors can improve microcirculatory dysfunction. However, the results of several clinical trials on the effects of SGLT2 inhibitors on coronary microcirculation have been different. Therefore, it is still lack of conclusive evidence on the effects of SGLT2 inhibitors on microcirculatory dysfunction. This review aims to summarize the completed and ongoing experiments regarding the effects of SGLT2 inhibitors on coronary microcirculation, in order to better elucidate the impact of SGLT2 inhibitors on microcirculation. It seeks to provide valuable information for the pharmacological mechanisms of SGLT2 inhibitors, the study of diseases related to coronary microcirculation disorders, and the treatment of heart failure.

## 1 Introduction

Heart failure is the end stage of various heart diseases and has become an important public health issue affecting the health of residents. Taking China as an example, the standardized prevalence rate of heart failure in the population aged 25 and above is 1.1%, which is approximately 12.1 million patients. The crude incidence rate is 248 cases per 100,000 people, with about 3 million new cases annually. The prognosis of heart failure patients is poor, with a mortality rate exceeding 50% in 5 years and a readmission rate of up to 69% within 1 year. Of those, 40.5% of the patients had more than 3 hospitalizations with an average hospitalization cost of 29,746 yuan and an average outpatient cost of 6,023 yuan per year (Wang et al., 2021; Mamas et al., 2017; Huang et al., 2017). In recent years, significant progress has been made in the treatment of management in heart failure, with the use of SGLT2 inhibitors being an important discovery in treatment (Zinman et al., 2015a; Neal et al., 2017a; Wiviott et al., 2019a; McMurray et al., 2019; Packer et al., 2020; Anker et al., 2021; Solomon et al., 2022). SGLT2 inhibitors has shown significant efficiency in several landmark heart failure randomized clinical trials and is recommended by major guidelines (Tsutsui, 2022; Heidenreich et al., 2022; Moghaddam et al., 2023; Patel et al., 2023). The simplicity, safety, and tolerability of SGLT2 inhibitors effectively combat clinical inertia in heart failure GDMT treatment (Khan et al., 2020), and it is widely used in the treatment of various types of heart failure. However, the full benefits of SGLT2 inhibitors on cardiovascular outcomes are not yet fully understood. In recent years, a series of studies have been conducted on the benefits of SGLT2 inhibitors in coronary microcirculation; however, the conclusions of these studies lack definitive opinions. This review will summarize the basic and clinical research on the effects of SGLT2 inhibitors on coronary microcirculation, which will help the development of heart failure treatment.

## 2 Coronary microcirculation and coronary microcirculatory diseases

### 2.1 Definition of coronary microcirculation

**FIGURE 1:**
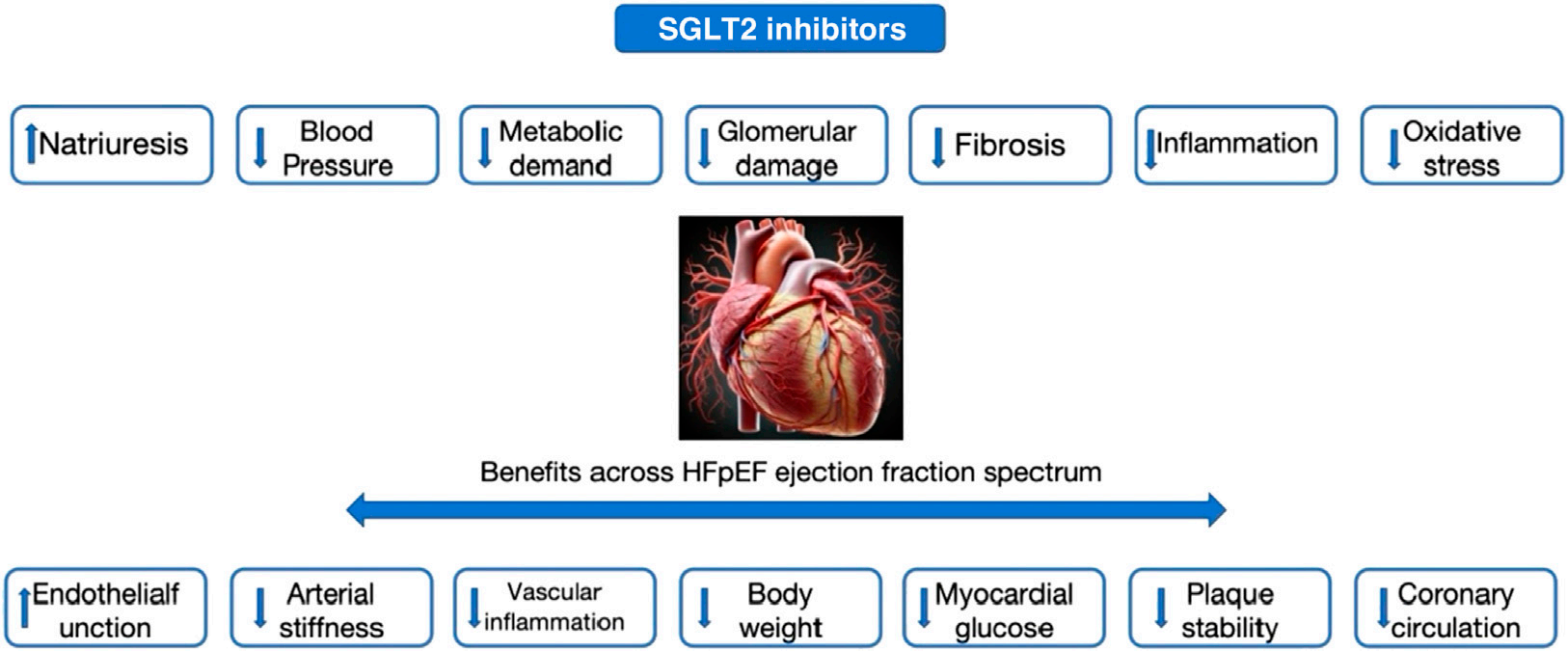
Benefits of SGLT2 inhibitors in HFpEF ejection fraction spectrum.

The coronary circulation is composed of epicardial coronary arteries (vessel diameter 0.5 mm–5 mm), arterioles (vessel diameter approximately 0.1 mm–0.5 mm), small arteries (vessel diameter <0.1 mm), and capillaries (vessel diameter <10um) (anatomy as shown in Figure 1). The arterioles, small arteries, and capillaries are the main components of the coronary microcirculation, which is responsible for regulating pressure and blood flow of the coronary arterioles. The arterioles are sensitive to pressure changes, while the distal arterioles are sensitive to flow changes. Capillaries provide 90% of the blood supply to the myocardium and serve as the sites for exchange of oxygen, nutrients, and metabolic substances in the myocardium. The coronary microcirculation plays an important role in regulating coronary perfusion pressure and blood flow through mechanisms of flow-mediated vasodilation, autoregulation of coronary blood flow, and regulation of myocardial oxygen consumption and metabolic substances (Chilian, 1997; Paulus and Tschope, 2013).

### 2.2 Evaluation indicator of coronary microcirculation

Coronary Flow Reserve (CFR) denotes the ratio of coronary blood flow (CBF) or myocardial blood flow (MBF) to the corresponding index at rest at or near maximal coronary artery dilation. It serves as a comprehensive indicator of the reserve function of the entire coronary artery system. CFR can be measured through PET, SPECT, cardiac magnetic resonance imaging (CMR), and coronary angiography. CFR is an independent predictor of major adverse cardiovascular events in patients with known or suspected coronary artery disease (CAD) (Indorkar et al., 2019). CFR is influenced by multiple factors, including coronary blood flow at rest (increased resting blood flow can lead to a decrease in CFR), the cross-sectional area of resistance vessels within a unit volume of myocardium (thickening of the vessel wall can lead to a decrease in CFR), the pressure outside the coronary vessels (increased wall tension can lead to a decrease in CFR), and coronary perfusion pressure (a decrease in blood pressure can lead to a decrease in CFR) (Marcus et al., 1987). Research findings indicate that in patients with coronary artery disease, if CFR <2.0, the 10-year major adverse cardiovascular event rate and cardiovascular mortality rate increase (Lee et al., 2022).

The index of microvascular resistance (IMR) in the coronary arteries is a new indicator proposed in recent years to evaluate the function of distal microvasculature in coronary artery stenosis. IMR is defined as the product of the pressure measured inside the coronary artery and the inverse of the mean transit time (Tm) in a hyperemic state. It reflects the specificity of myocardial microcirculation. Microvascular resistance is directly proportional to the pressure gradient across the myocardium (Pd-Pv) and inversely proportional to the blood flow (f) (Eshtehardi et al., 2011). In the absence of significant epicardial coronary artery stenosis, IMR has higher repeatability than CFR. IMR can specifically evaluate the function of microvasculature distal to the stenotic lesion and accurately predict myocardial tissue perfusion levels, ventricular remodeling, and recovery of cardiac function after acute myocardial infarction reperfusion therapy (Fearon et al., 2008; Ito et al., 2010). An IMR ≥25 is considered the threshold for diagnosing microvascular dysfunction (Lee et al., 2022).

The MBF refers to the small blood vessel network in the heart muscle, which is responsible for providing oxygen and nutrients to the myocardium. It responds to changes in the body’s metabolism and oxygen demand to meet the needs of the myocardial cells (Pelletier-Galarneau et al., 2019). MBF can be measured using Single-photon emission CT, PET, Transthoracic Doppler Echocardiography (TTDE), and CMR. The results of the above measurement are indicators of the quantity and velocity of the myocardial blood flow. Higher MBF usually indicates that the myocardial tissue is adequately supplied with blood to meet its metabolic demands, which is crucial for maintaining myocardial function. Conversely, lower MBF may indicate presence of coronary microvascular dysfunction or coronary artery disease.

Microvascular resistance reserve (MRR) is defined as the ratio of actual resting microvascular resistance to hyperemic microvascular resistance (HMR). The calculation is as follows: MRR = (Q_max_/Q_rest_) × (Pa,rest/Pd,hyper). Pa,rest represents the aortic pressure at rest, Pd,hyper represents the distal coronary artery pressure measured during hyperemia, while Q_rest_ and Q_max_ represents the actual blood flow measured at rest and during hyperemia, respectively. MRR can be invasively measured using coronary artery doppler, continuous thermodilution, or bolus thermodilution (De Bruyne et al., 2021).

HMR can measure the average flow velocity and pressure during the maximum hyperemic phase of the cardiac cycle in the distal coronary artery (or in the absence of stenotic lesions, in the distal coronary artery) using a doppler transducer and a pressure sensor mounted on a coronary guidewire. The calculation of HMR is as follows: HMR = pressure/flow rate (Xaplanteris et al., 2018). CFR <2.5 and HMR >1.7 mmHg/cm/s suggest coronary microvascular dysfunction. HMR is not affected by resting coronary blood flow.

Coronary Flow Velocity Reserve (CFVR) is typically assessed through TTDE or coronary angiography. CFVR represents the ratio of peak diastolic coronary artery blood flow velocity during hyperemia to the velocity at rest. Elevated CFVR value signals superior coronary microcirculation function and increased blood flow reserve, while decreased in CFVR may indicate presence of coronary microcirculation abnormalities, particularly prevalent in diabetic individuals (Jurgens et al., 2021).

### 2.3 Coronary microvascular disease

CMVD refers to the comprehensive effect of multiple pathogenic factors that lead to structural and/or functional abnormalities in the coronary pre-small arteries and small arteries resulting in clinical syndrome of effort angina or objective evidence of myocardial ischemia. Initially CMVD was named X Syndrome by Kemp HG in 1973, it was later referred to as microvascular dysfunction by Camici PG in 2007. In 2013, the European Society of Cardiology officially recognized it as microvascular dysfunction in stable coronary artery disease treatment guidelines, incorporated it into the spectrum of clinical coronary heart disease, and provided initial diagnostic and therapeutic recommendations (Montalescot et al., 2013). In 2017, the Chinese experts formally consented and designated it as “coronary microvascular disease” (Wenqiang et al., 2013).

The main pathophysiology of CMVD involves structural or functional abnormalities in the microvesses of the heart. Contributing factors include endothelial dysfunction, inflammatory responses, microvascular constriction, and platelet activation. These factors collectively result in compromised contraction and relaxation capabilities of the coronary microvessels, which impacts myocardial perfusion and metabolism. The aberrant microvascular function observed in CMVD patients can lead to myocardial ischemia and hypoxia with symptoms such as angina, myocardial ischemia, and potential myocardial damage. Furthermore, CMVD is closely linked to the occurrence of cardiovascular events and the progression of heart failure. Its involvement in the pathogenesis and progression of heart failure, diabetes, and coronary artery disease makes it a novel therapeutic target in these disease states.

Taking Heart Failure with Preserved Ejection Fraction (HFpEF) as an example, the PROMIS-HFpEF study confirmed the correlation between CMVD and HFpEF. The PROMIS-HFpEF study is a prospective, multicenter, multinational study aimed at investigating the prevalence and related factors of coronary microvascular dysfunction in patients with HFpEF (Shah et al., 2018). The study utilized echocardiography and adenosine-based transthoracic doppler echocardiography to measure CFR. The results showed that of the 202 HFpEF patients, 151 individuals (74.8%) had coronary microvascular dysfunction (CFR <2.5). Furthermore, in HFpEF patients, the prevalence of CMVD was high and was associated with atrial fibrillation, smoking, and abnormalities in certain physiological markers (i.e., urine albumin/creatinine ratio, troponin, and NT-proBNP levels). This study was recognized as one of the top ten cardiovascular disease advancements in 2018.

The pathogenesis of HFpEF is intricate with systemic inflammation playing a crucial role in its development (Fraction, 2023). Various conditions such as obesity, diabetes, chronic obstructive pulmonary disease, and salt-sensitive hypertension can trigger a systemic pro-inflammatory state leading to inflammation in the coronary microvascular endothelium. The above pathogenesis mechanism in turn, reduces the bioavailability of nitric oxide in neighboring myocardial cells, as well as the levels of cyclic guanosine monophosphate and protein kinase G (PKG) activity. The decrease in NO bioavailability and PKG activity promotes hypertrophic changes and increases resting tension in myocardial cells, contributing to myocardial stiffness and interstitial fibrosis, and ultimately leading to diastolic left ventricular (LV) stiffness and the progression of heart failure (Paulus and Tschope, 2013). Coronary microvascular dysfunction is initiated by a low-grade inflammatory response to cardiovascular risk factors, which results in local myocardial ischemia and hypoxia, and further exacerbate dysfunction in the coronary microvascular endothelium. Impaired coronary endothelial function leads to reduced microvascular NO bioavailability, as well as decreased levels of cGMP, PKG, and transforming growth factor (TGF)-β in myocardial cells, and ultimately promotes myocardial hypertrophy and fibrosis that contributes to the development of HFpEF (Paulus and Tschope, 2013; Fraction, 2023; van Heerebeek et al., 2012; Liu et al., 2017; Harvey et al., 2016; Forrester et al., 2018; Zile et al., 2015; Paulus, 2020; Schulz et al., 2008; Nikolic et al., 2022). The significant role of coronary microvascular dysfunction in the pathogenesis of HFpEF was highlighted in numerous studies (Table 1). (Paulus and Tschope, 2013; Shah et al., 2018; Lee et al., 2016; Taqueti et al., 2018; Mohammed et al., 2015; Sucato et al., 2015; Kato et al., 2016; Srivaratharajah et al., 2016; Dryer et al., 2018; Löffler et al., 2019; Kato et al., 2021; Ozcan et al., 2021; Ratchford et al., 2023; Arnold et al., 2022).

**TABLE 1:** Study on coronary microvascular dysfunction in patients with ejection fraction preserved heart failure.

First author	Sample size	Study design	Method	Outcome
Mohammed et al. (2015), Wang et al. (2021)	n = 124 HFpEF n = 104 controls	Retrospective, single center	Autopsy/pathology	Compared to controls, HFpEF patients had more coronary microvascular rarefaction and myocardial fibrosis, Microvascular density was inversely associated with myocardial fibrosis
Sucato et al. (2015), Mamas et al. (2017)	n = 155 HFpEF n = 131 non-HFpEF	Retrospective, single center	Invasive coronary angiography (TIMl frame count and TlMl myocardial perfusion grade)	HFpEF patients had worse TlMl frame count and worse TlMl myocardial perfusion grade in all three maior coronary artery territories compared to controls
Kato et al. (2016), Huang et al. (2017)	n = 25 HFpEF,n = 13 hypertensive LVH, and n = 18 controls	Prospective, single center	Cardiac MRl (CMD = CFR<2.5)	CMD prevalence in HFpEF = 76%: CFR was lowerin HFpEF compared with hypertensive LVH andcontrols; CFR correlated with BNP levels
Srivaratharajah et al. (2016), Zinman et al. (2015a)	n = 78 HFpEF,n = 298 non-HFpEF	Retrospective, single center	Rb-82 PET (CMD = MFR<2.0)	CMD prevalence in HFpEF = 40%; patients with HFpEF 2.6 times more likely to have CMD thancontrols even after adiustment for comorbidities
Taqueti et al. (2018), Neal et al. (2017a)	n = 201 without HFp EF (n = 36 with subsequent incident HFpEF)	Retrospective, single center	Rb-82 PET (CMD = MFR<2.0)	CMD was an independent risk factor for incident HFpEF; lower CFR was associated with worse LV diastolic function
Dryer et al. (2018), Wiviott et al. (2019a)	n = 30 HFpEF,n = 14 controls	Prospective, two center	Invasive coronary Doppler (CFR and lMR); CMD = CFR≤ 2.0 + IMR≥23)	CMD prevalence in HFpEF = 37% using CFR ≤2.0+IMR≥23; CMD prevalence in HFpEF = 47% using CFR <2.0; four-quadrant approach to defining CMD based on CFR and IMR
Shah et al. (2018), McMurray et al. (2019)	n = 202 HFpEF	Prospective, multicenter, multinational	Echo/Doppler CFR(CMD = CFR< 2.5)	CMD prevalence in HFpEF = 75%.CMD patients were more likely to have a history of atrial fibrillation and smoking, CFR correlated with multiple indices including UACR, NT-proBNP, RHI, TAPSE, RV, LV, and LA strain
Löffler et al. (2019), Packer et al. (2020)	n = 19 HFpEF,n = 15 controls	Prospective, single center	CMR (MVD = MPR <2.5)	Compared to controls, HFpEF patients had reduced global MPR (2.29 ± 0.64 vs. 3.38 ± 0.76, p = 0.002).The prevalence of CMD in the HFpEF group was 69%
Kato et al. (2021), Anker et al. (2021)	n = 163 HFpEF	Retrospective, single center	CMR (CMD = CFR< 2.0)	On a Kaplan Meier curve, the rates of adverse events were significantly higher in HFpEF patients with CFR <2.0 compared with HFpEF with CFR ≥2.0 (p < 0.001)
Ozcan et al. (2021), Solomon et al. (2022)	n = 80 (n = 18AF; n = 40 HFpEF)	Prospective, single center	ICPS(CMD = CFR< 2.0)	Patients with AF (61%) and HFpEF (62%) or both (71%) are more likely to develop abnormal CFR than patients without these conditions
Stephen (2022), Tsutsui (2022)	n = 14 HFpEF	Prospective, single center	PLM procedure/Doppler	Compared with the control group, brachial artery RH was significantly reduced by 30% in patients with HFpEF, compared with a similar decrease in the RH index, which was independently associated with future cardiovascular events
Arnold et al. (2022), Heidenreich et al. (2022)	n = 101 HFpEF	Prospective, single center	Transthoracic echocardiography/CMR (MVD = MPR <2.0)	MPR was lower in patients with HFpEF *versus* control subjects (1.74 ± 0.76 vs. 2.22 ± 0.76; P < 0.001). MVD was present in 70% of patients with HFpEF (vs. 48% of control subjects; P < 0.014)

Abbreviations: AF, atrial fibrillation; CMD, coronary microvascular dysfunction; LV, left ventricular; LA, left atrial; RHI, reactive hyperemia index; RH, reactive hyperemia; TAPSE, tricuspid annular plane systolic excursion; UACR, Urinary albumin-to-creatinine ratio; MPR, myocardial perfusion reserve; ICPS, invasive coronary physiology studies.

In conclusion, CMVD is involved in the occurrence and development of various diseases, making it an important therapeutic target. Therefore, effective intervention in coronary microvascular dysfunction is crucial for the prevention and treatment of cardiovascular diseases and their related complications.

## 3 Sodium-glucose cotransporter 2 inhibitors and their effects on coronary microcirculation

### 3.1 Benefits of sodium-glucose cotransporter 2 inhibitors in heart failure

SGLT2 inhibitors are a class of anti-diabetic drugs. With ongoing research, the benefits of SGLT2 inhibitors in heart failure are becoming increasingly significant. Clinical studies have accumulated evidence from patients with diabetes and without diabetes, from HFrEF to HFpEF, and from chronic to acute heart failure. This shift from being primarily antidiabetic agents to heart failure treatment drugs has been incorporated into guidelines, providing a new tool for improving the prognosis of heart failure patients. Studies such as EMPA-REG OUTCOME, CANVAS, and DECLARE-TIMI58 have shown the cardiovascular safety of SGLT2 inhibitors in diabetic patients (Zinman et al., 2015b; Neal et al., 2017b; Wiviott et al., 2019b), particularly in reducing the risk of heart failure hospitalization. Trials like DAPA-HF, EMPEROR-Reduced, EMPEROR Preserved, and DELIVER have demonstrated the ability of SGLT2 inhibitors to reduce the risk of heart failure hospitalization and cardiovascular death in HFrEF, HFpEF, and HFmrEF (McMurray et al., 2019; Packer et al., 2020; Anker et al., 2021; Solomon et al., 2022). The SOLOIST-WHF study confirmed the benefits of SGLT2 inhibitors in patients with worsening of heart failure. SGLT2 inhibitors have become the first class of heart failure treatment drugs to span across all ejection fractions (Bhatt et al., 2021).

The 2021 ESC included SGLT2 inhibitors for the first time in the international authoritative heart failure guidelines, listing them as cornerstone medications for heart failure with reduced ejection fraction. It emphasized that SGLT2 inhibitors, as one of the four cornerstone drugs, should be used in combination early on. The 2022 ACC guidelines recommended SGLT2 inhibitors as a Class 2A recommendation for treating patients with heart failure with preserved ejection fraction. In August 2022, the Chinese Heart Failure Center Alliance Expert Committee established the Chinese expert consensus on the clinical application of SGLT2 inhibitors in heart failure to standardize their rational use. The consensus recommended a clinical application pathway for using SGLT2 inhibitors in heart failure. The 2023 update of the Chinese National Heart Failure Guidelines highlighted SGLT2 inhibitors as a first-line choice for treating HFpEF and HFmrEF (Fraction, 2023).

The expression of SGLT2 is highly specific to renal tissue and is expressed in pancreatic alpha cells, but not in human heart. Only SGLT-1 is expressed at a low level (Di Franco et al., 2017). Therefore, the potential effects of SGLT-2 inhibitors on cardiac function may be indirect, mainly mediated by systemic hemodynamics and metabolism (Lee et al., 2017). In the rat model of myocardial infarction, SGLT-2 receptor seems to reduce the release of myocardial superoxide, the presence of myofibroblasts and inflammatory macrophages, and subsequent myocardial fibrosis. In diabetic mice, SGLT-2 inhibition decreased the expression of transforming growth factor-β, the levels of type I and III collagen and overall cardiac fibrosis (Li et al., 2019). It is not clear whether this effect also occurs in humans.

Studies in structural and functional research have shown that SGLT2 inhibitors can improve cardiac structure progression by reducing left ventricular mass, inhibiting left ventricular wall thickness, and slowing the progression of left ventricular wall thickness and cavity radius (Verma et al., 2019; Soga et al., 2018; Brown et al., 2020; Matsutani et al., 2018). SGLT2 inhibitors mainly improve cardiac function by increasing LVEF and reducing left ventricular end-diastolic volume, left ventricular end-systolic volume, left atrial volume index, and left ventricular mass index (Lee et al., 2021; Lan et al., 2021; von Lewinski et al., 2022; Ersbøll et al., 2022).

Diastolic dysfunction is the core hemodynamic feature of ejection fraction retention (HFpEF) in heart failure, which involves many physiological processes, including left ventricular remodeling, cardiac metabolic dysfunction and extracellular matrix fibrosis. In animal models and patients of HFpEF, reduced bioavailability of endothelium-dependent nitric oxide (NO) has been shown to promote the proliferation of fibroblasts and myofibroblasts and affect the diastolic function of energy-dependent cardiomyocytes by regulating the low phosphorylation of cytoskeletal protein Titin (Borbély et al., 2009). Studies from endomyocardial biopsy samples from patients with HFpEF have shown that inflammatory microvascular endothelial cells contribute to the migration of monocytes and promote the release of transforming growth factor-β (TGF--β), which promotes the transformation, collagen production and cross-linking of fibroblasts to myofibroblasts (Mohammed et al., 2015; Borbély et al., 2009; Kirkman et al., 2020). In addition, this pro-inflammatory and pro-oxidative environment may make dysfunctional coronary microvessels more prone to recurrent myocardial ischemia and microinfarction, further leading to interstitial fibrosis, changes in substrate metabolism and decreased systolic reserves, resulting in the occurrence of HFpEF (Mohammed et al., 2015). Research in pathophysiology has shown that the effects of SGLT2 inhibitors on cardiomyocyte hypertrophy and cardiac fibroblast fibrosis are mainly achieved through the regulation of multiple signaling pathways, including the AMPK/mTOR pathway, JAK/STAT pathway, SGK1 pathway, and sGC/cGMP/PKG pathway, leading to a reduction in cardiomyocyte volume increase; lowering of ventricular mass index; reduction in the extent of cardiac fibrosis and decrease in the deposition of extracellular matrix in cardiomyocytes, thereby inhibiting cardiomyocyte hypertrophy and non-cardiomyocyte proliferation, reducing ventricular mass increase and cardiac fibrosis (Yurista et al., 2019; Li et al., 2021; Hsieh et al., 2022; Lin et al., 2022; Xue et al., 2019). Additionally, SGLT2 inhibitors also improve cardiac structure and function by regulating cellular pathophysiological processes such as autophagy, apoptosis, oxidative stress, mitochondrial function, energy metabolism, angiogenesis, and signaling pathway dysregulation (Li et al., 2021; Nah et al., 2020; Liu et al., 2021; Long et al., 2022; Xing et al., 2021; Bugga et al., 2022). These regulatory effects help alleviate inflammation and cellular dysfunction during the process of cardiac remodeling (Figure 1).

SGLT2i has shown a wide range of benefits in cardiovascular treatment, including its potential effects on coronary microcirculation function. Coronary microcirculation disturbance plays an important role in the development of diabetes and cardiovascular disease. However, the effect of SGLT2 inhibitors on coronary microcirculation has not been determined. Several animal experiments and clinical studies have explored the effects of SGLT2 inhibitors on Coronary microcirculation disturbance, but the results are different and lack of a conclusive opinion. This review aims to summarize the current research and provide valuable insights for the pharmacological mechanism of SGLT2 inhibitors and the pathological mechanism of CMVD.

### 3.2 Effect of SGLT2 inhibitors on coronary microcirculation: Animal studies

L-arginine is known to be the physiological precursor of NO formation in endothelium-dependent vasodilation. NO has a variety of intracellular effects, which can lead to vasodilation, endothelial regeneration, inhibition of leukocyte chemotaxis and platelet adhesion. In a study conducted by Adingupu et al., in 2020, db/db mice were treated with Englergin for 10 weeks and then used high-resolution ultrasound imaging to measure CFVR to evaluate coronary microcirculation. Among them, SGLT 2i increased the bioavailability of NO by increasing L-arginine/ADMA ratio, and then improved CFVR and myocardial flow reserve (Adingupu et al., 2019). In 2022, Yimin Tu et al. randomly divided db/db mice into db/db group, db/db + EMPA group, and db/m mice as control group. After 8 weeks of treatment, it was found that EMPA could inhibit the loss of cardiac pericytes and increase the coverage of pericytes to coronary microvessels in type 2 diabetic mice (Tu et al., 2022). In previous studies, it has been found that the decrease of the number and coverage of cardiac pericytes may be involved in the progress of CMD (Ferland-McCollough et al., 2017), in which pericytes play an important role in maintaining vascular stability, and the improvement of their number and function is helpful to improve the function and structure of cardiac microvessels (Trost et al., 2016). These animal experimental results support the view that SGLT2 inhibitors improve coronary microcirculation in animal models (Table 2).

**TABLE 2:** Animal study of SGLT2 Inhibitors on coronary artery microcirculation.

Study (year)	Drug/Dosage	Animals	Duration	Measuring tools	Measured index	Outcome
Adingupu et al. (2019), Wang et al. (2021)	Empagliflozin (1.5 mg/kg/day)	21	10 weeks	Transthoracic echocardiography	CFVR	SGLT2 Inhibitors improves coronary microvascular function and contractile performance
Tu et al. (2022), Mamas et al. (2017)	Empagliflozin (10 mg/kg/day)	26	8 weeks	Echocardiographic	CFV CFR	Empagliflozin attenuated coronary microvascular function and structural abnormalities and protected cardiac pericytes in diabetic mice

Abbreviations: CFVR, coronary flow velocity reserve; CFR, coronary flow reserve; CFR, coronary flow reserve.

### 3.3 Effect of SGLT2 inhibitors on coronary microcirculation: clinical studies

Multiple clinical trials, however, have found a lack of conclusive evidence on the impact of SGLT2 inhibitors on coronary microcirculation.

In 2021, Katrine M et al. conducted a randomized double-blind, placebo-controlled crossover trial on 12 patients with a 4-week treatment of empagliflozin and placebo. Evaluation using 11C-acetate PET/CT after the experiment showed that empagliflozin reduced resting MBF by 13%, which remained significant even after adjusting for cardiac workload, but had no significant effect on stress MBF. On the whole, SGLT2 inhibitors is a benign result for coronary microcirculation disturbance (Lauritsen et al., 2021). In 2022, a preliminary report by Leccisotti et al. on the DAPAHEART Trial demonstrated the random allocation of 16 patients to receive dapagliflozin (n = 8) or placebo (n = 8) for 4 weeks in a double-blind, placebo-controlled treatment. Evaluation using FDG PET/CT after the experiment showed a significant improvement in myocardial flow reserve in patients receiving dapagliflozin treatment. Resting rate-pressure product correction confirmed an increase in myocardial flow reserve in the dapagliflozin group. Resting MBF in the dapagliflozin group was significantly lower than in the placebo group, even after correcting for resting rate-pressure product (Leccisotti et al., 2022). The above studies have found that SGLT2 inhibitors can improve the disturbance of coronary microcirculation.

However, a randomized, placebo-controlled crossover study conducted by Lauritsen et al., in 2021 found that after 12 weeks of treatment with empagliflozin 25 mg and placebo, there was no significant impact on CFVR assessed by TTDE (Suhrs et al., 2022). Additionally, a randomized study consistent with this result investigated 90 patients with Type 2 Diabetes and cardiovascular disease or high risk, and found that empagliflozin treatment for 13 weeks had no effect on myocardial flow reserve measured by Rubidium-82 positron emission tomography (Jurgens et al., 2021). In 2022, Magnus Lundin et al. conducted a randomized trial on sodium-glucose cotransporter inhibition (SOCOGAMI) in patients with newly discovered glucose abnormalities and recent myocardial infarction, where 42 patients were randomized to receive empagliflozin 25 mg (n = 20) or placebo (n = 22), and after 7 months of treatment, measurements using CMR and echocardiography showed that empagliflozin did not affect coronary flow reserve (Lundin et al., 2022).

Based on current clinical research, the conclusions regarding the efficacy of SGLT2 inhibitors on coronary microcirculation in actual clinical populations are not consistent. This phenomenon reflects that SGLT2 inhibitors may exhibit differentiated effects in different patient populations and disease characteristics. Therefore, more large-scale, long-term clinical trials are needed in the future to further validate their efficacy on coronary microcirculation in different patient groups, in order to comprehensively assess their true value in clinical practice. A prospective, single-center, randomized LUCENT-J study is currently underway in Japan (Tamanaha et al., 2024), which is a prospective, single-center, randomized, two-arm, parallel-level, active-controlled study. Forty Type 2 Diabetes patients are being randomly assigned in a 1:1 ratio to receive dapagliflozin or the control group and treated for 24 weeks. Changes in myocardial flow reserve are measured using 13N-ammonia positron emission tomography computed tomography scans, and the experimental results will provide more clinical evidence for us to observe the efficacy of SGLT2 inhibitors on coronary microcirculation (Table 3).

**TABLE 3:** Clinical study of SGLT2 Inhibitors on coronary artery microcirculation.

Study (year)	Drug/Dosage	Patients	Duration	Measuring tools	Measured index	Outcome
Katrine et al. (2020), Wang et al. (2021)	Empagliflozin (25 mg/day)	12	4 weeks	11C-acetate PET/CT	MFR MBF	EMPA reduced resting MBF by 13%, which was significant even after adjustment for cardiac workload. But did not significantly affect stress MBF.
Leccisotti et al. (2022), Mamas et al. (2017)	Dapagliflozin (10 mg/day)	16	4 weeks	FDG PET/CT	MBF MFR	SGLT2 Inhibitors improved resting MBF corrected for cardiac workload in T2D patients to improvement the coronary microvascular dysfunction
Lauritsen et al. (2021), Huang et al. (2017)	Empagliflozin (25 mg/day)	26	12 weeks	TTDE	CFVR	There was no significant effect on the primary outcome, CFVR, after empagliflozin treatment nor placebo
Jurgens et al. (2021), Zinman et al. (2015a)	Empagliflozin (25 mg/day)	90	13 weeks	82Rb-PET/CT	MFR	Empagliflozin did not improve CFVR in patients with DM2
Lundin et al. (2022), Neal et al. (2017a)	Empagliflozin (25 mg/day)	42	10 months	CMR and Echocardiography	CFR	Coronary flow reserve was not affected by Empagliflozin
Tamanaha et al. (2024), Wiviott et al. (2019a)	Luseogliflozin (2.5 mg/day)	40	24 weeks	13N-ammonia PET	MFR	Ongoing

Abbreviations: CFVR, coronary flow velocity reserve; CFR, coronary flow reserve; IMR, index of microvascular resistance; MBF, myocardial blood flow; MRR, Microvascular resistance reserve; TTDE, transthoracic doppler echocardiography; CMR, Cardiac Magnetic Resonance; MFR, myocardial flow reserve.

## 4 Conclusion

The current clinical studies on the efficacy of SGLT2 inhibitors on coronary microcirculation in real clinical populations yield inconsistent conclusions. This may be related to the design of the study, the differences among the subjects and the diversity of measurement methods. Therefore, further large-scale, long-term clinical trials are needed to comprehensively evaluate the exact effect of SGLT2 inhibitors on coronary artery microcirculation in different patient groups, so as to determine the best clinical application and potential benefits. These results are expected to provide useful clues for the pharmacological study of SGLT2 inhibitors, especially for the mechanism of coronary microcirculation.
